# Effect of autogenous tooth vs. autogenous bone grafts on periodontal phenotype around immediate implants: A randomized clinical trial

**DOI:** 10.34172/japid.026.4099

**Published:** 2025-12-02

**Authors:** Adileh Shirmohammadi, Elnaz Ziaei-Rad, Leila Roshangar, Samira Mohammad Mirzapour, Fatemeh Aghaziarati

**Affiliations:** ^1^Department of Periodontics, Faculty of Dentistry, Tabriz University of Medical Sciences, Tabriz, Iran; ^2^Department of Anatomical Sciences, Faculty of Histology and Embryology, Tabriz University of Medical Sciences, Tabriz, Iran

**Keywords:** Autografts, Bone regeneration, Dental implants, Randomized controlled trial

## Abstract

**Background.:**

Immediate implant placement can result in a horizontal defect between the implant and alveolar bone, potentially affecting osseointegration and esthetic outcomes. Grafting this space supports bone regeneration. This study compared the effectiveness of autogenous tooth-derived and autogenous bone grafts for horizontal gap augmentation in the maxillary anterior region.

**Methods.:**

This parallel-arm randomized, single-masked clinical trial enrolled patients attending the Tabriz University of Medical Sciences School of Dentistry, who were candidates for immediate implant placement in the maxillary anterior region. After implant placement, the control sites (n=11) received autogenous bone grafts. In contrast, the intervention sites (n=11) received autogenous dentin grafts to fill the horizontal gap between the implant and the buccal wall (jumping gap). A connective tissue graft was subsequently placed in both groups to enhance buccal soft tissue. Buccal bone thickness (measured 2 mm and 5 mm below the marginal crest) and soft tissue thickness were measured at two points at the time of surgery and again six months postoperatively. Data were analyzed using paired t-tests to assess changes within each group and independent t-tests to compare differences between the two groups.

**Results.:**

Both groups showed significant increases in buccal bone and soft tissue thickness compared with baseline. At 2 mm below the marginal crest, buccal bone thickness did not differ significantly between the two groups (*P*=0.401). At 5 mm below the marginal crest, the autogenous bone graft group showed significantly greater bone thickness (2.15±0.21 mm) than the dentin graft group (1.50±0.53 mm, *P*=0.001). There was no significant difference in buccal soft tissue thickness changes between the bone graft (1.15±0.18 mm) and dentin graft (1.1±0.17 mm) groups.

**Conclusion.:**

Both autogenous dentin and bone grafts effectively enhanced buccal bone and soft tissue thickness around immediate implants. Autogenous bone grafts produced significantly greater increases in buccal bone at 5 mm below the marginal crest. **Trial Registration.** Iranian Registry of Clinical Trials (IRCT), IRCT20110726007128N11, Trial ID 86072.

## Graphical Abstract

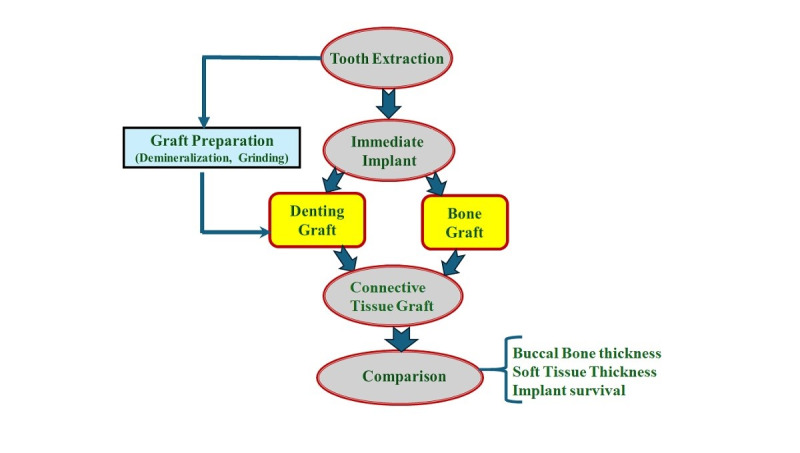

## Introduction

 Replacing missing teeth with implant-supported prostheses is an established treatment modality.^1^ Traditionally, dental implants are placed following a healing period of approximately six months after tooth extraction, as proposed by Brånemark in the 1980s, to ensure complete hard and soft tissue healing and successful osseointegration.^2^ However, the requirement for full post-extraction healing has been challenged, giving rise to immediate implant placement protocols.^3-5^ Immediate implant placement refers to inserting an implant into the extraction socket immediately after tooth removal.^6^ This approach minimizes surgical interventions and shortens the overall treatment time.^7^ Despite these advantages, immediate implantation can increase the risk of esthetic complications and infection.^8-10^ Following extraction, the alveolar socket often exceeds the implant diameter, resulting in a horizontal gap between the implant surface and surrounding bone, known as the “jumping gap.”^11^ Filling this space is particularly critical on the buccal aspect in esthetic regions, where the cortical plate is typically thin and susceptible to resorption, which can compromise soft tissue contours.^12-14^ Gaps < 2 mm may heal without grafting, but larger gaps require bone augmentation to ensure optimal outcomes.^15-17^

 Conventional graft materials have limitations. Autogenous bone grafts are considered the gold standard due to their osteogenic potential; however, they are limited by donor site morbidity and insufficient volume.^18^ Allografts and xenografts avoid secondary surgery but may exhibit unpredictable resorption, slow remodeling, or ethical constraints. Alloplastic materials are biocompatible but generally lack bioactivity.^19,20^ These limitations underscore the need for alternative biologically favorable graft materials. Autogenous dentin grafts have emerged as a promising option due to their osteoconductive and osteoinductive properties, their compositional similarity to bone, and their lack of immune reactions.^21-25^ Demineralized dentin matrix provides a scaffold rich in collagen, BMPs, and growth factors, promoting new bone formation while maintaining alveolar ridge volume.^25,26^ The dentin matrix also acts as a biocompatible scaffold with slow resorption, providing a favorable environment for osteoblast adhesion and proliferation. These properties help maintain bone volume and reduce marginal bone resorption, thereby playing an important role in the success of implant osseointegration.^22^ Histologic studies demonstrate that demineralized dentin grafts can integrate effectively with the host bone, promote new bone formation, and support long-term implant stability.^26-29^ Radiographically, demineralized dentin grafts have been associated with the preservation of the buccal and palatal cortical plates, increased alveolar ridge thickness, and minimal marginal bone loss over long-term follow-ups (mean follow-up: 3–5 years), confirming both structural and functional stability around implants.^30-32^ Despite these advantages, clinical data on the use of autogenous dentin for gap management in immediate implants remain limited.^33^ This study aimed to evaluate the clinical and radiographic outcomes of autogenous dentin grafts for horizontal gap management following immediate implant placement in esthetic zones, addressing whether dentin grafts can serve as a safe, effective, and reliable alternative to conventional graft materials.

 The null hypothesis of this study was that there would be no significant difference between autogenous dentin grafts and autogenous bone grafts in preserving buccal bone and soft tissue thickness following immediate implant placement.

## Methods

###  Population and study design

 The study population consisted of all patients presenting to the Implant Department of the Tabriz University of Medical Sciences School of Dentistry, who were candidates for implant placement in the maxillary anterior region (canine to canine). Twenty-two teeth from these patients, meeting the inclusion criteria, were included in the study. Of these, 11 teeth were assigned to the test group and 11 to the control group. The selection ensured comparable baseline characteristics between the groups and enabled evaluation of the clinical and radiographic outcomes of autogenous dentin versus autogenous bone grafts in immediate implant placement. All the patients were fully informed about the potential risks, possible complications, and anticipated benefits of the treatment. Information was provided both orally and in writing, and written informed consent was obtained from all the participants. The surgical procedures were conducted in accordance with the principles of the Declaration of Helsinki of 1975, as revised in 2008. The study protocol was approved by the Ethics Committee of Tabriz University of Medical Sciences (IR.TBZMED.DENTISTRY.REC.1403.072). The trial was registered in the Iranian Registry of Clinical Trials (IRCT) under the code (IRCT20110726007128N11).

 The patients were eligible for the study if they met all of the following criteria: adults aged > 18; systemic health suitable for oral surgery (ASA I or II); healthy periodontium in adjacent teeth, defined as no probing depth ≥ 4 mm and no bleeding on probing; clinical indication for immediate implant placement in the maxillary anterior region for endodontic or restorative reasons; and the ability and willingness to attend follow-up visits and provide written informed consent. Patients were excluded from the study if they presented any of the following conditions: poor oral hygiene, defined as a high plaque index or inability to maintain oral hygiene; active smoking ( > 10 cigarettes/day or ≥ 10 pack-years); pregnancy or breastfeeding; severe periodontal disease; uncontrolled diabetes (HbA1c > 7.0%); history of head and neck radiotherapy; current or past use of bisphosphonates, denosumab, or long-term systemic corticosteroids or other immunosuppressants; alcohol or substance abuse; systemic or metabolic bone disorders affecting healing; inadequate bone anatomy that would prevent achieving primary implant stability; inability or unwillingness to attend follow-up visits; or active infection at the extraction site, indicated by clinical signs of infection or purulence.

 Based on a previous similar clinical trial by Elfana et al,^34^ the means ± standard deviations of bone thickness for demineralized autogenous dentin grafts and autogenous bone grafts were 48.4 ± 11.5 mm and 37.5 ± 9 mm, respectively. Considering a type I error of < 5% (α < 0.05) and a power of 80%, the required sample size was estimated at 11 teeth per group, yielding a total of 22 teeth.

###  Randomization

 In this study, the participants were allocated in a 1:1 ratio using block randomization with a block size of 4 to either the intervention or control group. Randomization was performed at the individual level. The random sequence was generated using statistical software (SPSS Random Number Generator). To ensure allocation concealment, sequentially numbered, opaque, sealed envelopes were used. These envelopes were prepared and safeguarded by an independent colleague who was not part of the research team. After obtaining informed consent and confirming eligibility criteria, the envelope corresponding to the patient’s inclusion order was opened to reveal the assigned intervention type. Outcome assessors measuring buccal bone and soft tissue thickness were blinded to group allocation to minimize measurement bias.

###  Autogenous dentin graft preparation

 The tooth-crushing technique has been widely proposed as a method for preparing autogenous dentin grafts. Initially, this technique was primarily applied to freshly extracted third molars, with radiographic and clinical evaluations at six months postoperatively, demonstrating natural bone regeneration following grafting using bone mills and hammers.^35^ For this study, extracted teeth were selected based on the patient’s treatment plan, with extraction indicated for periodontal or prosthetic reasons. After extraction, all soft tissue, debris, calculus, and root cementum were removed using a sickle scaler, and the pulp tissue within the root canal was removed with a K-file. The enamel was removed using a turbine bur, leaving the dentin intact. The remaining dentin was rinsed with sterile saline and placed into a bone mill, producing particles ranging from 1 to 1.5 mm in size. The particles were then washed with 1.0-M sodium chloride solution and partially demineralized by immersion for 10 minutes in 2% nitric acid (pH = 1.0), followed by 10 minutes in 0.1-M Tris–HCl solution (pH = 7.4). To decellularize the samples, they were treated with trypsin and carbohydrates (raffinose and sucrose) in Hank’s balanced buffer. Finally, the particles were washed three times for 10 minutes each in a penicillin/streptomycin-containing PBS solution on a shaker incubator and disinfected using UV irradiation.

###  Surgical procedure

 Patients who met the inclusion criteria were selected from those referred to the Implant Department. For each patient, a cone-beam computed tomography (CBCT) scan was obtained preoperatively as a baseline reference, and all the surgical procedures were performed by the same experienced surgeon. The surgical field was prepared under sterile conditions using 5% povidone-iodine for disinfection and sterile draping. Before surgery, each patient rinsed with 0.2% chlorhexidine gluconate mouthwash for 30 seconds to reduce the microbial load. Following local anesthesia, tooth extraction was performed atraumatically, and the integrity of the socket walls was evaluated. Patients with buccal bone fractures or dehiscence at the extraction site were excluded from the study. Any residual granulation tissue was carefully removed using a curette.

**Figure 1:**
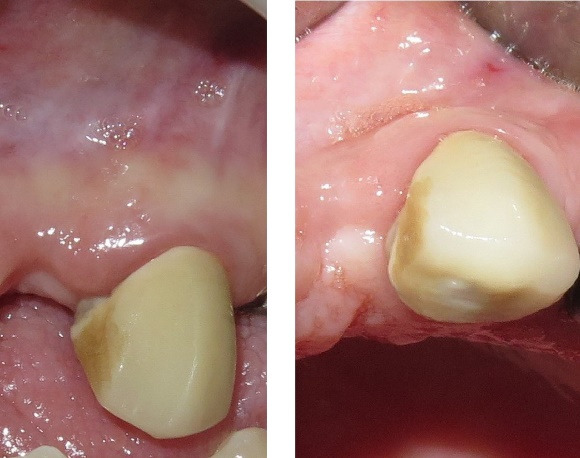
Preoperative view

**Figure 2:**
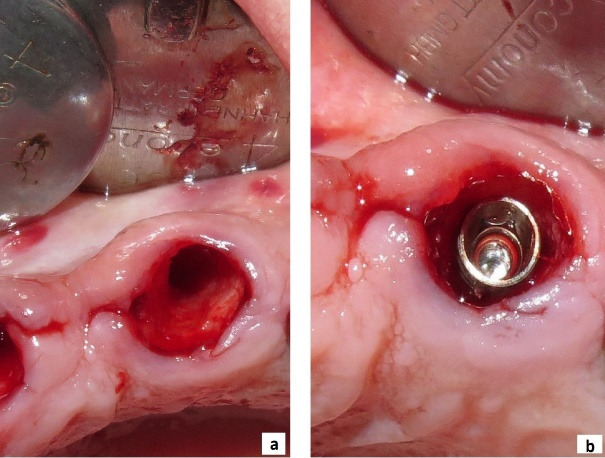
a) Atraumatic extraction. b) Implant placement

**Figure 3:**
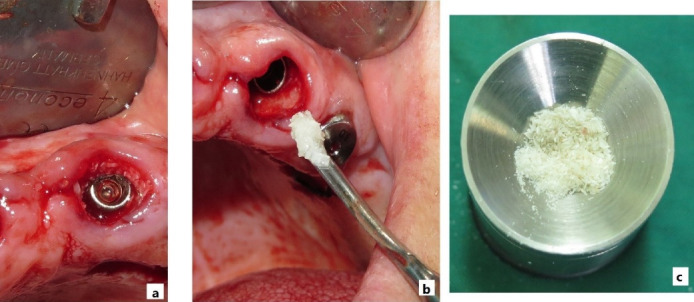
a) Placement of autogenous dentin graft. b, c) Autogenous denting graft

**Figure 4:**
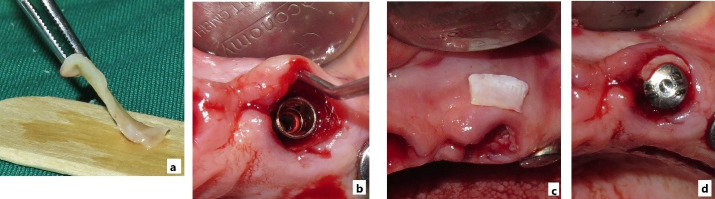
a) Connective tissue graft. b) Creation of a facial pouch. c, d) Insertion of a subepithelial connective tissue graft

 The final implant dimensions were determined after evaluation of the socket anatomy. Sequential drilling was performed under copious irrigation until the desired implant dimensions were achieved. To obtain optimal primary stability, the implant was placed 2–3 mm beyond the apex of the extracted tooth and 1–2 mm below the crestal bone level. The horizontal gap (jumping gap) between the implant surface and the buccal bone wall was measured using a periodontal probe to confirm that it exceeded 2 mm. The patients were then randomly assigned to one of the two groups. In the test group, the gap was filled with autogenous dentin graft particles prepared as described above and condensed around the implant. In the control group, the gap was filled with autogenous bone graft material. In both groups, a subepithelial connective tissue graft was used to enhance buccal soft tissue thickness. A facial pouch was carefully prepared without damaging the mesial or distal papillae. A 1.5-mm-thick subepithelial connective tissue graft of appropriate dimensions was harvested from the palate using a scalpel, and the epithelial layer was removed with a 15C blade. The graft was then sutured at the gingival margin level (Figures 1 to 4).^36^

###  Radiographic measurements

**Figure 5:**
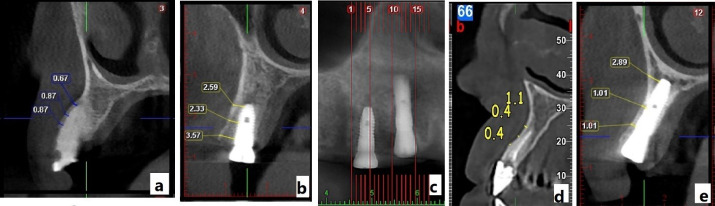
a) Pre-extraction radiographic assessment. b) Radiographic assessment of autogenous denting graft six months after surgery. c) CBCT evaluation. d) Pre-extraction radiographic assessment. e) Radiographic assessment of autogenous bone graft six months after surgery

 CBCT was used for radiographic assessment before surgery and six months after implant placement in both groups. All the CBCT scans were performed at the same radiology center using identical imaging parameters to ensure consistency. The radiographic evaluation focused on measuring changes in buccal bone thickness at two reference points—2 mm and 5 mm apical to the marginal crest—both before implant placement and six months postoperatively. All the measurements were performed by a calibrated examiner who was blinded to the group allocation to minimize assessment bias. Calibration was conducted before the study by having the examiner measure a subset of scans twice, one week apart, to ensure consistent measurements. The results obtained from both groups were then compared statistically (Figure 5).

###  Soft tissue thickness measurement

**Figure 6:**
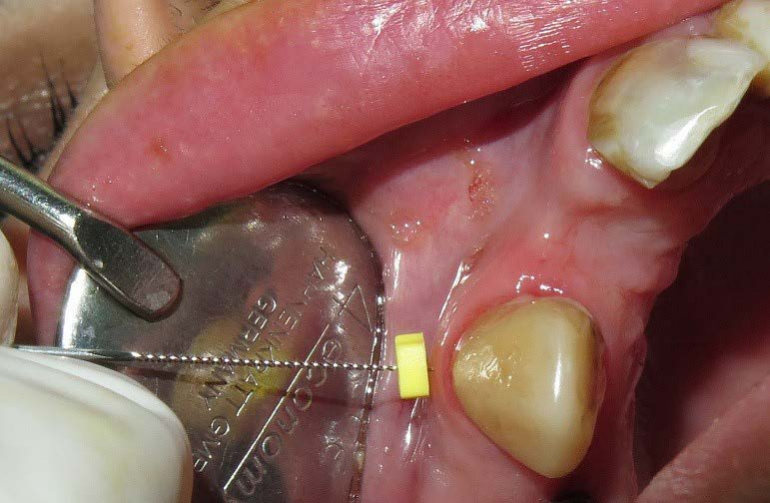
Assessment of soft tissue thickness

 To evaluate the buccal soft tissue thickness, a #20 endodontic file was gently inserted perpendicularly through the mucosa until a firm resistance indicating contact with the underlying bone was felt. A silicone stopper was then positioned flush with the mucosal surface, and the file was carefully withdrawn. The distance from the tip of the file to the base of the silicone stopper was measured using a ruler with 0.1-mm divisions to obtain precise soft tissue thickness values. Measurements were taken at the midfacial point, 3 mm apical to the line connecting the gingival margins of the two adjacent teeth. Baseline measurements were performed at the time of implant placement under local anesthesia (Figure 6).^37^

###  Implant survival 

 Implant survival was evaluated based on clinical and radiographic criteria. The parameters included the presence of the implant in function, the absence of radiolucency around the implant on radiographic examination, the absence of recurrent or persistent peri-implant infection, and the absence of pain, discomfort, or paresthesia reported by the patient in the corresponding area.^38^

## Results

 All implants in both groups met the success criteria outlined above throughout the observation period. No biological complications such as peri-implantitis or infection were observed in either group during follow-up.

###  Radiographic assessment

 After six months, the mean buccal bone thicknesses at 2 mm below the marginal crest were 1.773 ± 0.186 mm in the control group and 1.808 ± 0.807 mm in the test group. In both the test and control groups, bone density increased significantly from baseline (Table 1).

 To compare the buccal bone thickness at 2 mm below the marginal crest between the test and control groups, the difference in buccal bone thickness before surgery and six months after implant placement was calculated for each group. The mean difference in the control group was 0.98 ± 0.16 mm, whereas it was 1.20 ± 0.85 mm in the test group. Although the mean increase in the test group was higher than in the control group, the independent t-test revealed no statistically significant difference between the two groups (*P* = 0.401) (Table 2).

 After six months, the mean buccal bone thickness at 5 mm below the marginal crest was 2.95 ± 0.95 mm in the control group and 2.64 ± 0.74 mm in the test group. Both groups showed a statistically significant increase in bone thickness compared to baseline values (Table 3).

 To compare buccal bone thickness between the groups, the difference between baseline and six-month measurements was calculated. The mean increase in the bone graft group (2.15 ± 0.21 mm) was significantly greater than that in the dentin graft group (1.50 ± 0.53 mm), and the independent t-test revealed a statistically significant difference between the two groups (*P* = 0.001) (Table 4).

###  Buccal soft tissue thickness

 The mean buccal soft tissue thicknesses at 6 months were 2.1364 ± 0.1685 mm in the control group and 2.1000 ± 0.14142 mm in the test group. Both groups showed a significant increase in soft tissue thickness compared with baseline (Table 5).

 The mean increase in gingival thickness was 1.15 ± 0.18 mm in the bone graft group and 1.1000 ± 0.17321 mm in the dentin graft group. Independent t-test revealed no statistically significant difference between the two groups (*P* = 0.485) (Table 6).

**Table 1:** Buccal bone thickness at 2 mm below the margin in the bone graft and dentin groups (paired-samples t-test)

**Variable**	**N**	**Mean (mm)**	**Standard deviation**	**T**	**df**	***P*** ** value**
Bone graft (pre)	11	0.8000	0.1265			
Bone graft (post)	11	1.7773	0.1862	-20.631	10	0.000
Dentin graft (pre)	11	0.6082	0.2137			
Dentin graft (post)	11	1.8082	0.8078	-4.707	10	0.001

**Table 2:** Comparison of differences in buccal bone thickness at 2 mm below the margin between the bone graft and dentin graft groups (independent t-test)

**Group**	**N**	**Mean difference (mm)**	**Standard deviation**	**T**	**Df**	***P*** ** value**
Bone graft	11	0.9773	0.1571			
Dentin graft	11	1.2000	0.8455	-0.859	20	0.401

**Table 3:** Buccal bone thickness at 5 mm below the margin in the bone graft and dentin graft groups (paired-samples t-test)

**Variable**	**N**	**Mean (mm)**	**Standard deviation**	**T**	**df**	***P*** ** value**
Bone graft (pre)	11	0.8000	0.0775			
Bone graft (post)	11	2.9545	0.2067	-33.788	10	0.000
Dentin graft (pre)	11	0.5718	0.2064			
Dentin graft (post)	11	2.0745	0.6375	-9.429	10	0.000

**[Table T4]. **

**Table 4 T4:** Comparison of differences in buccal bone thickness at 5 mm below the margin between the bone graft and dentin graft groups (independent t-test)

**Group**	**N**	**Mean difference (mm)**	**Standard deviation**	**T**	**df**	***P*** ** value**
Bone graft	11	2.1545	0.2115			
Dentin graft	11	1.5027	1.5027	3.7970	20	0.001

**Table 5:** Buccal soft tissue thickness in the bone graft and dentin graft groups (paired-samples t-test)

**Variable**	**N**	**Mean (mm)**	**Standard deviation**	**T**	**df**	***P*** ** value**
CTG GT (pre, bone graft)	11	0.9818	0.0982			
CTG GT (post, bone graft)	11	2.1364	0.1079	-20.5480	10	0.000
CTG GT (pre, dentin graft)	11	0.9727	0.1104			
CTG GT (post, dentin graft)	11	2.1000	0.1414	-20.8410	10	0.000

**Table 6:** Comparison of buccal soft tissue thickness between the bone graft and dentin graft Groups (independent-samples t-test)

**Group**	**N**	**Mean (mm)**	**Standard deviation**	**t**	**df**	***P*** ** value**
Bone graft	11	1.1545	0.1864			
Dentin graft	11	1.1000	0.1732	0.7110	20	0.4850

## Discussion

 This study assessed buccal bone thickness changes at 2 mm (T_1_) and 5 mm (T_2_) from the marginal crest in patients receiving autogenous bone grafts or autogenous dentin grafts. Both grafts significantly increased buccal bone thickness after six months (*P* < 0.001). At T_1_, the mean increase was 0.98 ± 0.18 mm for autogenous bone grafts and 1.20 ± 0.84 mm for dentin grafts, with no significant difference (*P* > 0.05). At T_2_, autogenous bone grafts showed a significantly greater increase (2.15 ± 0.21 mm) compared to dentin grafts (1.50 ± 0.52 mm) (*P* = 0.001).

 This superiority can be attributed to the osteogenic potential of bone grafts, which contain viable osteoblasts, osteoclasts, and progenitor cells that actively participate in remodeling and integration within the deeper, cancellous bone regions, characterized by higher vascularity and trabecular structure that supports rapid cellular infiltration and new bone formation. In contrast, dentin grafts, being acellular, primarily function through osteoconduction (providing a scaffold) and osteoinduction (releasing growth factors like BMPs), but depend on host cell migration for remodeling, which may be less efficient in apical zones due to slower resorption rates and limited immediate cellular contribution. The marginal 2 mm area (T_1_), predominantly cortical bone, experiences resorption pressures from mechanical loading and periosteal disruption that are similar to those in the graft, thereby mitigating graft-specific advantages. In contrast, the 5 mm depth benefits more from the bone grafts’ ability to enhance volume preservation and stability in spongy bone. These results align closely with prior studies employing similar autogenous materials.

 In the study by Wu et al,^39^ horizontal bone changes around 30 immediate implants in the anterior maxilla were evaluated at 0, 3, and 6 mm from the marginal crest during 6- and 12-month follow-up periods. The results showed that horizontal bone reduction in the autogenous dentin graft group was almost comparable to that of the xenograft group at all measurement levels and time intervals, indicating similar stability between the two graft materials. However, bone loss at the 6-mm level was significantly less than at the 0- and 3-mm levels, suggesting that the more apical regions of the bone are more stable and less prone to resorption than the marginal areas. These findings are consistent with the results of the present study, in which buccal bone thickness increased in both groups after six months. Notably, in the bone graft group, the increase was significantly greater at the deeper level (T_2_, 5 mm below the marginal crest). This similarity in apical stability may be related to the biological characteristics of bone in this region, which is less susceptible to resorption due to its greater distance from superficial mechanical forces and its denser structure. However, a critical comparison must consider a key difference in the surgical technique: the present study used a flapless approach, whereas Wu et al^39^ employed a full-thickness mucoperiosteal flap. The full-thickness flap disrupts periosteal blood supply, potentially increasing osteoclastic activity and leading to greater bone resorption, especially in marginal regions. In contrast, the flapless approach used in the present study preserves the periosteum and improves blood supply, likely contributing to increased thickness at both 2- and 5-mm levels below the crest. This technical difference may explain why the present study reported gains in bone thickness, whereas Wu et al^39^ focused on the extent of bone resorption. In other words, preserving blood supply in the flapless approach may have promoted more active bone formation in the present study, while the full-thickness flap in Wu’s study possibly enhanced marginal bone loss, showing relative stability only in the deeper regions.

 Similarly, Cinar et al^40^ retrospectively analyzed 110 immediate implants and reported that autogenous mineralized dentin grafts (AMDG) preserved buccal thickness better than deproteinized bovine bone (Bio-Oss®) at 5 mm in the anterior maxilla (*P* < 0.05), though initial bone thickness influenced outcomes. Overall, the findings indicate that autogenous mineralized dentin grafts (AMDG) preserve buccal bone thickness more effectively than Bio-Oss. When comparing our findings to those of Cinar et al,^40^ a key difference is the type of grafts evaluated. Cinar et al’s study compared autogenous dentin grafts with xenografts derived from an external source, which might have elicited an immune response or slower integration, whereas our study compared two autogenous grafts (bone and dentin) harvested from the same patient, which share similar biological properties. This difference may explain why AMDG showed superior performance compared with Bio-Oss® in Cinar et al’s study, whereas in our study autogenous bone grafts demonstrated greater increases in buccal bone thickness, particularly at the 5-mm level below the crest, possibly due to their stronger osteogenic potential compared with the primarily osteoconductive dentin grafts.

 Abd Dkheel and Al-Quisi^41^ further corroborated dimensional stability with autogenous dentin grafts in 20 immediate anterior implants, showing alveolar ridge volume was maintained within a range similar to the preoperative condition (*P* = 0.567). In both this study and the present research, a flapless approach was employed, as previous studies have shown that flap elevation can disrupt the periosteum, reduce blood supply to the buccal bone, and increase osteoclastic activity, ultimately leading to greater buccal bone resorption compared to the flapless technique. Therefore, the flapless approach in both studies possibly contributed to better preservation of buccal bone volume.

 Regarding implant survival, our study achieved 100% survival in both groups over six months (*P* > 0.05), with no radiolucent areas, persistent infections, or patient complaints. This aligns with Wu et al,^39^ who reported 100% survival for 30 immediate implants at 12 months, Cinar et al,^40^ who confirmed 100% survival in 110 implants with minimal prosthetic complications, and Abd Dkheel and Al-Quisi,^41^ who observed 100% success in 20 implants, emphasizing esthetic outcomes, though our six-month follow-up limits long-term extrapolations. Minetti et al^27^ reported a 98.2% survival rate with dentin grafts at one year, suggesting sustained viability.

 The findings of the present study have important clinical implications for implant therapy in the esthetic zone, particularly in patients with thin buccal bone phenotypes. Maintaining or increasing buccal bone thickness is critical to achieving long-term esthetic outcomes, as thin buccal bone is more prone to resorption and soft tissue recession.^42^ Our results suggest that both autogenous dentin and bone grafts can effectively preserve buccal bone dimensions, with bone grafts showing superior increases at deeper levels. These findings highlight the potential of autogenous grafts to enhance implant stability, support soft tissue contours, and reduce the risk of esthetic compromise, making them valuable tools in treatment planning for high-risk esthetic cases.

## Limitations

 One of the main limitations of this study is the small sample size (11 cases per group), which may have reduced statistical power and increased the risk of a type II error. This could explain the lack of significance observed for some differences. However, the normality of the data distribution was confirmed using the Kolmogorov-Smirnov test (*P* > 0.05), justifying the use of the t-test and maintaining the reliability of the analyses under the current conditions. Also, the six-month follow-up limits insights into long-term bone remodeling and implant stability, potentially overlooking late resorptions. Nonetheless, it is recommended that future studies be conducted with larger sample sizes (at least 30–50 cases per group) based on precise power calculations, as well as with longer follow-up periods, to substantially improve the generalizability and robustness of the results.

## Conclusion

 Within the limitations of this randomized clinical trial, both autogenous bone and dentin grafts improved buccal bone and soft tissue thickness around immediate implants in the maxillary anterior region. Autogenous bone grafts yielded greater buccal bone gain at 5 mm below the marginal crest. Further long-term and histological studies are required to determine the clinical significance of these dimensional differences.

## Competing Interests

 Dr. Adileh Shirmohammadi was the Editor-in-Chief of the Journal of Advanced Periodontology & Implant Dentistry (JAPID) at the time of publication of this paper. The authors declare no other competing interests concerning authorship and/or publication of this article.

## Data Availability

 The datasets used and/or analyzed during the current study are available from the corresponding author upon reasonable request.

## Ethical Approval

 The study protocol and informed consent forms were approved by the Research Ethics Committee of Tabriz University of Medical Sciences (approval no: IR.TBZMED.DENTISTRY.REC.1403.072). All the participants provided written informed consent forms before being included in the study.
